# Safety and Efficacy of Rituximab in Multiple Sclerosis: A Retrospective Observational Study

**DOI:** 10.1155/2018/9084759

**Published:** 2018-11-12

**Authors:** Bassem I. Yamout, Nabil K. El-Ayoubi, Johny Nicolas, Yehya El Kouzi, Samia J. Khoury, Maya M. Zeineddine

**Affiliations:** ^1^Nehme and Therese Tohme Multiple Sclerosis Center, American University of Beirut Medical Center, Beirut, Lebanon; ^2^Faculty of Medicine, American University of Beirut, Lebanon

## Abstract

**Objective:**

To evaluate the efficacy and safety of rituximab in multiple sclerosis in a clinical practice setting.

**Methods:**

Clinical data for all adult patients with multiple sclerosis (MS) treated with off-label rituximab at a single MS center in Lebanon between March 2008 and April 2017 were retrospectively collected from medical charts. The main efficacy outcomes assessed were annualized relapse rate (ARR) and proportion of patients free from relapses, disability progression, or magnetic resonance imaging (MRI) activity.

**Results:**

A total of 89 rituximab-treated patients were included: 59 relapsing-remitting MS (RRMS) and 30 progressive MS (PMS). Patients were treated with 1000 or 2000 mg rituximab IV every 6–12 months for a mean duration of 22.2 ± 24.8 months. The subjects were 65.2% females with a mean age of 40.5 ± 12.3 years and a mean disease duration of 7.9 ± 6.2 years. During treatment, the ARR decreased from 1.07 at baseline to 0.11 in RRMS (*p* < 0.0001) and from 0.25 to 0.16 in PMS patients (*p* = 0.593). The mean Expanded Disability Status Scale (EDSS) remained unchanged in both RRMS and PMS patients. Between baseline and the last follow-up, the percent of patients free from any new MRI lesions increased from 18.6% to 92.6% in the RRMS group and from 43.3% to 82% in the PMS group. No evidence of disease activity (NEDA) was achieved in 74% of patients at 1 year of treatment. A total of 64 adverse events (AEs) (71.9%) were recorded with the most common being infusion-related reactions in 25.8% of patients, all mild in nature. Two of our rituximab-treated patients experienced serious AEs requiring surgical interventions: pyoderma gangrenosum vaginalis with perianal abscess and fistula and an increase in the size of a meningioma. No case of progressive multifocal leukoencephalopathy (PML) was detected.

**Conclusion:**

In our real-world cohort, rituximab was well-tolerated and effective in reducing relapse rate and disability progression in relapsing-remitting and progressive MS patients.

## 1. Introduction

There is increasing evidence that B cells and humoral immunity play a key role in the pathogenesis of multiple sclerosis (MS) [1]. Rituximab is an anti-CD 20 chimeric monoclonal antibody that effectively depletes circulating B cells [2]. B cell depletion is expected to alter B cell-mediated antigen presentation and subsequent T cell activation, antibody production, and cytokine secretion. A randomized placebo-controlled phase II trial of rituximab in relapsing-remitting MS (RRMS) demonstrated robust efficacy on clinical and radiological outcomes, with an acceptable safety profile [3]. A phase III trial of rituximab in primary progressive multiple sclerosis (PPMS) did not meet its primary efficacy endpoint, but subgroup analysis showed delayed disability progression in younger patients (age < 51 years) with enhancing (Gd+) lesions on magnetic resonance imaging (MRI) [4]. In March 2017, the US Food and Drug Administration (FDA) approved ocrelizumab, a humanized anti-CD 20 monoclonal antibody, to treat adult patients with RRMS and PPMS. Ocrelizumab was shown to be effective and safe in two phase III trials conducted in RRMS patients with a significant reduction in annualized relapse rate (ARR), which confirmed disability progression and new MRI lesions [5]. In the ORATORIO trial [6], ocrelizumab reduced the rate of disability progression in patients with PPMS compared to placebo. Ofatumumab, a fully humanized anti-CD 20 monoclonal antibody, was recently shown in a phase II placebo-controlled trial to reduce new Gd + lesions by 65% [7]. All 3 monoclonal antibodies deplete CD20 B cells but differ in their chimeric (rituximab), humanized (ocrelizumab), or fully humanized (ofatumumab) molecular composition. Rituximab is frequently used off-label in MS, although its efficacy, safety profile, and dosing schedule are not well characterized for MS therapy [8]. The aim of this study was to assess the efficacy and safety of rituximab in patients with relapsing-remitting or progressive MS followed at a specialized academic MS center (MSC) in Lebanon.

## 2. Methods

### 2.1. Study Design and Population

This retrospective cohort study was conducted using data from a single MS center registry at the American University of Beirut Medical Center (AUBMC). All patients were captured from prospectively collected data through an established registry with a protocol that includes an EDSS assessment and brain MRI every 6–12 months. The study was approved by the Institutional Review Board of AUBMC.

All patients diagnosed with MS according to the 2010 revised McDonald criteria [9] and ever treated with rituximab with ≥3 months of follow-up were included in this study. The exclusion criteria were (1) patients treated with rituximab for other concomitant medical conditions and (2) lack of follow-up data.

### 2.2. Treatment Protocols

Patients were usually treated with an initial higher loading dose (2000 mg intravenous (IV) rituximab subdivided into 2 infusions given at 2-week interval) then maintained on single infusions of 1000 mg every 6–12 months. Peripheral blood CD-19 cell counts were not used to guide treatment decisions.

### 2.3. Data Collection

We reviewed the charts of all MS patients treated with rituximab with ≥3 months of follow-up between March 2008 and October 2017. Data collected included age, gender, disease duration, treatment duration, previous therapies, reason for switching to rituximab, MS phenotype, clinical relapses, Expanded Disability Status Scale (EDSS), and adverse events (AEs). Clinical examinations and MRI scans were obtained as part of routine practice every 6–12 months after initiation of any new disease-modifying therapy and new T2 and/or gadolinium enhancing (Gd+) lesions were assessed compared to a baseline scan performed within 3 months before the initiation of rituximab. All scans were performed using 1.5 or 3 Tesla magnet MRI machines pre- and postgadolinium injection.

The main efficacy outcomes were as follows: (1) proportion of patients free from relapses, (2) ARR, (3) proportion of patients free from disability progression (as evidenced by EDSS), (4) proportion of patients free from new T2 and/or Gd + lesions on brain MRI, and (5) proportion of patients with NEDA defined as the absence of relapses, disability progression, and new/Gd + lesions on brain MRI. All AEs occurring during rituximab treatment were also recorded.

### 2.4. Statistical Analyses

Statistical analysis was performed using the Statistical Package for Social Sciences software (IBM SPSS Statistics for Windows, version 25.0. Armonk, NY: IBM Corp.). The statistical significance was defined as a two-sided *p* value *<* 0.05. The normality of distributions was evaluated through the Shapiro-Wilk test. Data was reported as the mean (standard deviation) and median (range) or counts (proportions) for continuous and categorical variables, respectively. The relapse rate over the period of 2 years prior to the initiation of rituximab (pre-treatment) and after the last follow-up (posttreatment) was calculated. Comparisons between pre- and posttreatment relapse rates and EDSS were performed using the nonparametric Wilcoxon signed-rank test for paired samples, analyzing these changes separately among the group of patients with RRMS and progressive MS (PMS). We lumped patients with primary and secondary progressive MS together as a single group of PMS as per the Lublin phenotypes [10]. We defined progressive relapsing MS (PRMS) or progressive MS with activity (Lublin phenotype) as continuous disability progression independent of relapses with ≥1 relapse during the preceding year.

Bivariate analyses were performed to explore associations between different variables at baseline and response to treatment (NEDA), reporting Pearson's correlation coefficients and *p* values. Multivariable logistic regression (stepwise forward regression) was performed to build a model of the baseline predictors of NEDA after at least 1 year of treatment with rituximab, reporting odds ratios (OR) and 95% confidence intervals (CI). Due to the exploratory nature of the study, no adjustment for multiple comparisons was performed.

## 3. Results

**Figure 1:**
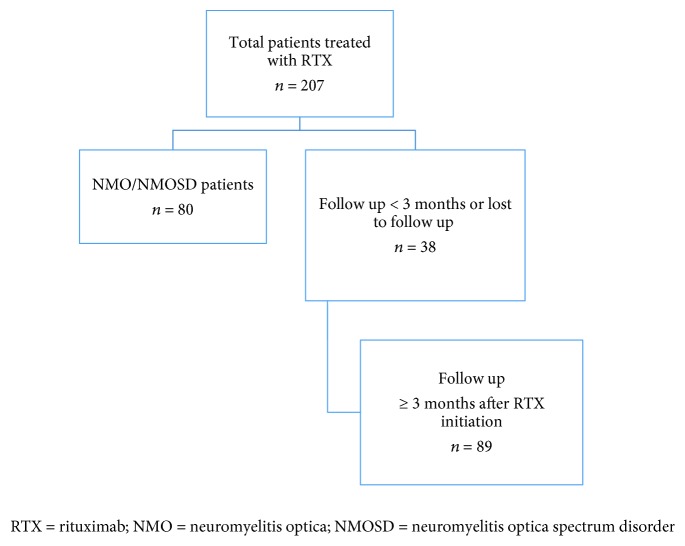
Cohort selection: RTX = rituximab, NMO = neuromyelitis optica, and NMOSD = neuromyelitis optica spectrum disorder.

A total of 207 patients received rituximab at our center, of whom 80 were diagnosed with neuromyelitis optica. Of the remaining 127 patients with MS, 38 had a posttreatment follow-up of <3 months. The remaining 89 patients (59 RRMS and 30 PMS) fulfilled the inclusion criteria and were included in the study (Figure 1). Baseline characteristics of the 89 patients are reported in Table 1. The 30 patients with PMS included 2 with PPMS, 8 with PRMS, and 20 with SPMS. Among those, 9/30 patients had a total of 15 relapses in the preceding 2 years.

The mean age and disease duration of our patients were 40.5 ± 12.3 years and 7.9 ± 6.2 years, respectively, and fifty-eight (65.2%) were females. Only 10 (11.2%) patients received rituximab as their first disease-modifying drug (DMD), while all the others switched from other therapies, mainly interferon-beta (IFN) and fingolimod (Table 1). The most common reason for switching to rituximab was persistent disease activity on other DMDs (87.3% of patients). Sixty-eight patients (76.4%) received an initial loading dose of 2000 mg followed by the maintenance of 1000 mg every 6 months. The mean treatment duration with rituximab was 22.2 ± 24.8 months (median 13 [3–113]). The mean pretreatment EDSS was 3.61 ± 1.95 (median 3.5 [0–6.5]), and 36% of patients had enhancing lesions on their baseline MRI.

**Figure 2:**
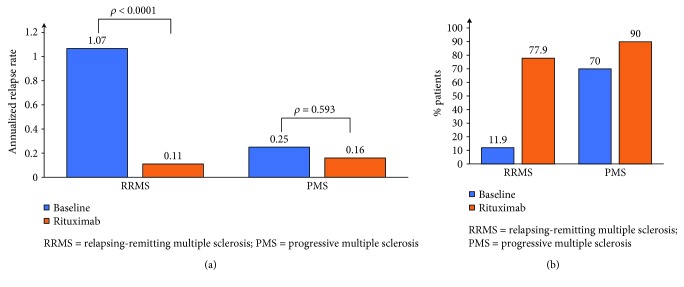
Annualized relapse rate (a) and proportion of patients free from relapses (b).

**Figure 3:**
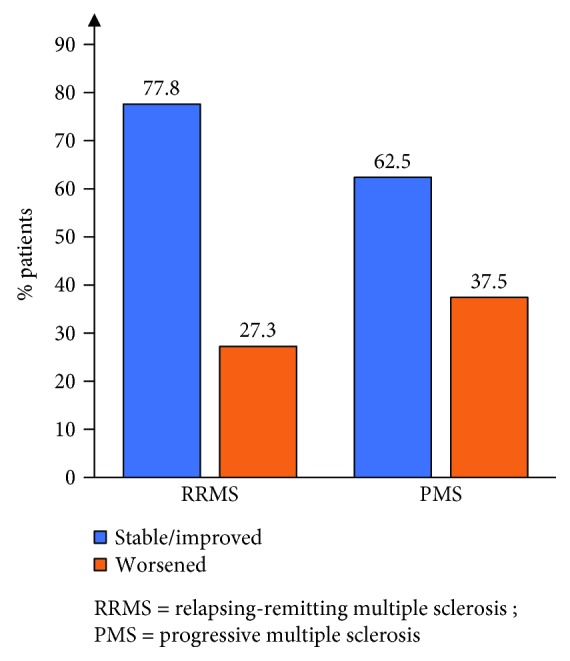
Proportion of patients with EDSS changes on rituximab.

**Figure 4:**
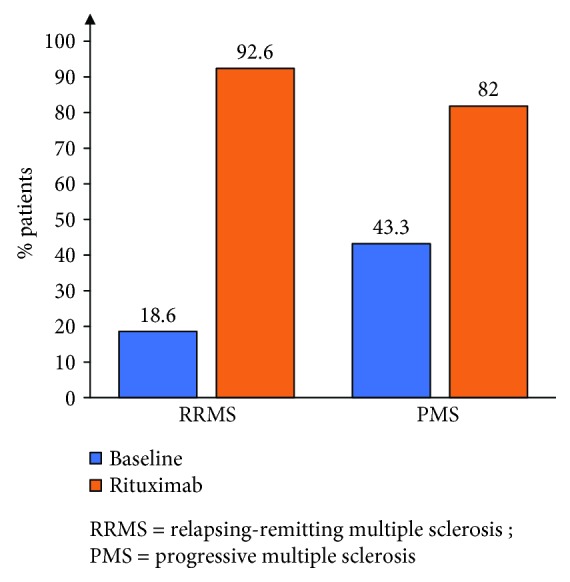
Proportion of patients free of new/Gd + lesions.

Clinical disease activity during rituximab therapy was low in both the RRMS and the PMS groups. The ARR dropped from 1.07 ± 0.8 (median 1 [0–4]) to 0.11 ± 0.26 (median 0 [0–1]) in the RRMS group (*p* < 0.0001) and from 0.25 ± 0.43 (median 0 [0–1.5]) to 0.16 ± 0.74 (median 0 [0–4]) in the PMS group (*p* = 0.593) (Figure 2(a)). A total of 77.9% and 90.0% of patients were relapse-free in the RRMS and the PMS groups, respectively (Figure 2(b)). Out of 15 relapses on rituximab in the whole cohort, 8 occurred in the first 6 months including 3 within 1 month of treatment initiation. The proportion of patients free from disability progression, as assessed by EDSS, was 77.8% in the RRMS group and 62.5% in the PMS group (Figure 3). There was a trend of improving EDSS during rituximab therapy compared to baseline in RRMS patients: 2.89 ± 1.62 (median 3 [0–6.5]) pretreatment vs 2.77 ± 2.02 (median 2.5 [0–7.5]) posttreatment (*p* = 0.05). In PMS patients, there was no significant change in EDSS: 5.25 ± 1.59 (median 6 [2–6.5]) pretreatment vs 4.91 ± 1.96 (6 [1.5–8]) posttreatment (*p* = 1.0). Similarly, radiological activity was significantly reduced during rituximab therapy. Follow-up MRIs were available for 54/59 RRMS and 22/30 PMS patients. At baseline, only 18.6% of RRMS and 43.3% of PMS patients were free of new/Gd + lesions, while 92.6% of RRMS and 82% of PMS patients had no new/Gd + lesions during treatment (Figure 4). At baseline, 13.3% of PMS patients had Gd + lesions on MRI. Only 3.9% of patients showed Gd + lesions during rituximab treatment as opposed to 36% at baseline. In patients with at least 12 months of follow-up postrituximab, 83.7%, 91.3%, and 92.3% were free of relapses, disability progression, and new/Gd + lesions, respectively, at 1 year, and their ARR at 1 year was 0.16. NEDA was achieved in 74% (40/54) of patients at 1 year (68.7% in PMS and 76.3% in RRMS) and 76% of patients (38/50) at 2 years (72.7% in PMS and 76.9% in RRMS).

In bivariate analyses, the following variables were not significantly associated with NEDA after 1 or 2 years of treatment: gender (*p* = 0.21), age (*p* = 0.15), disease duration (*p* = 0.96), number of DMDs prior to the initiation of rituximab (*p* = 0.16), number of relapses in the previous 2 years before starting rituximab (*p* = 0.12), months between the latest relapse and first rituximab infusion (*p* = 0.58), presence of brain or spine MRI activity in the year prior to rituximab initiation (*p* = 0.31), and first cycle of rituximab of 2000 mg as loading dose (0.89). There was a trend for the type of MS course (*p* = 0.06), with higher rates of NEDA achieved in RRMS vs PMS patients. However, baseline EDSS before treatment initiation was the only variable negatively correlated with maintaining NEDA at 1 year (*r* = −0.27, *p* = 0.011) and 2 years (*r* = −0.37, *p* = 0.006) of treatment. In multivariate analyses and after controlling for age, gender, disease duration, presence of MRI activity in the brain or spine before rituximab initiation, and receiving the first induction cycle of 2000 mg, a lower baseline EDSS was still associated with NEDA after one year (OR = 0.75, 95% CI = 0.59–0.96, *p* = 0.027) and 2 years of treatment with rituximab (OR = 0.63, 95% CI = 0.4–0.99, *p* = 0.045).

**Figure 5:**
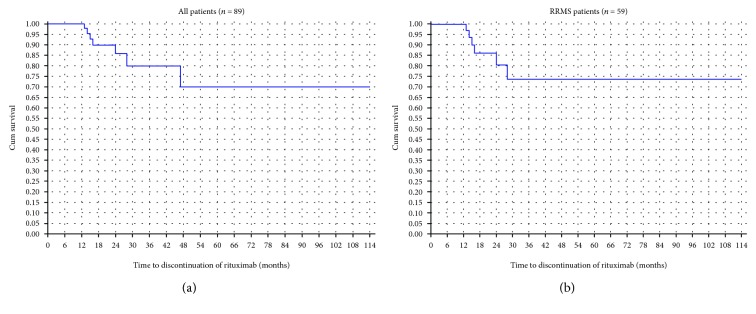
Survival analysis graph with the outcome of “drug discontinuation due to relapses, disability progression, and adverse events” (a) among all patients in the rituximab cohort and (b) among RRMS patients.

Rituximab infusions were generally well tolerated: 23 patients (25.8%) experienced a total of 40 infusion-related AEs all of which were mild and self-limited. A total of 64 (71.9%) AE occurred on rituximab, most of which were mild to moderate. Besides infusion-related reactions, the most common AEs were infections (*n* = 14) (Table 2). Two patients (2.2%) developed serious adverse events. A 29-year-old woman developed 38 months after initiating rituximab severe fungal vaginal infection, pyoderma gangrenosum vaginalis, and perianal abscess with fistula formation requiring a colostomy. Another 42-year-old woman with a stable convexity meningioma for the last 6 years had 21 months into treatment, sudden growth in size, new enhancement, and cystic changes of the tumor, requiring surgical resection. The pathology was consistent with an atypical meningioma. This sudden change in the tumor behavior was attributed to repeated cycles of hormonal stimulation for in vitro fertilization rather than rituximab therapy, although a role for B cell-depleting therapies in tumor induction cannot be ruled out. Serum immunoglobulins were not monitored routinely in our center at the time, but our single patient with pyoderma gangrenosum had low IgG (4.64 g/L) and normal IgM (0.52 g/L) serum levels. Rituximab treatment was discontinued in 17 patients (19.1%): 11 with RRMS and 6 with PMS. The main reason for discontinuation was lack of efficacy as evidenced by new relapses or disability progression (*n* = 13), while only 1 patient stopped treatment due to adverse events. In the remaining 3 patients, drug discontinuation was due to pregnancy, lack of reimbursement, and personal preference. In the PMS group, all 6 patients discontinued treatment due to worsening EDSS without superimposed relapses after a mean duration of 26.6 months (range 5–46). In the RRMS group, 7 patients discontinued treatment due to inefficacy after a mean duration of 16.9 months (range 6–38), all of them due to worsening disability, with conversion to SPMS in 4 and a superimposed relapse in the second year of treatment in 1 patient. The drug survival (patients not discontinuing drug due to relapses, disability progression, or adverse events) was around 85% at 2 years (Figure 5). There were no cases of progressive multifocal leukoencephalopathy (PML) although only 31/89 patients were checked for JC virus antibodies. Out of those, 27 patients tested were positive but only 14/27 received rituximab for more than 2 years.

## 4. Discussion

We report our experience with the off-label use of rituximab in MS patients followed at a single center in the Middle East for a mean of 22.2 months. Rituximab was mostly used as a second-line therapy in RRMS patients with suboptimal response to the first-line DMDs and PMS patients with evidence of progressive disability: 88.8% of patients were previously treated with at least 1 DMD and 87.3% of switchers did so due to lack of efficacy of previous DMDs. In this population, rituximab was highly effective in suppressing relapses in both RRMS (ARR = 0.11) and PMS (ARR = 0.16) patients and in preventing disability progression with 77.8% and 62.5% of patients with RRMS and PMS, respectively, showing no evidence of disability progression on treatment. This effect on disability progression was also reflected by a stable EDSS all through the treatment period for both RRMS and PMS patients with a trend to improve EDSS in the former. Rituximab was also highly effective in suppressing disease activity on MRI: 92.6% and 82% of patients with RRMS and PMS, respectively, showed no new and/or Gd + lesions during the treatment period. Only 3.7% of patients on rituximab developed Gd + lesions on MRIs as opposed to 36% at baseline before treatment initiation. The high relapse rate and number of Gd + lesions at baseline in our PMS cohort are due to the fact that 8/30 patients had PRMS and therefore were in the transition phase between RRMS and SPMS but with definite evidence of disability progression independent of relapses. This reflects our treatment approach of early use of rituximab in patients with progressive disease, which we think might improve the final outcome. Finally, 74% and 76% of patients achieved NEDA at 1 and 2 years, respectively. In a multivariate analysis, EDSS and to a lesser extent the type of MS were the best predictors of response, which translates into, as in all other MS therapies, starting treatment early before the accumulation of disability in patients with RRMS failing first-line therapies and as soon as the transition from RRMS is clinically evident in patients with SPMS.

Since the original phase II HERMES trial by Hauser et al. [3] showed robust efficacy of rituximab in MS based on clinical and radiological parameters, the off-label use of rituximab gained significant ground within the international MS community. This was further encouraged by the recent FDA and EMA approval of ocrelizumab as the first B cell-depleting therapy in MS. The obvious reasons for such practice are the similar mechanisms of action of rituximab and ocrelizumab as they both deplete B cells by binding to the CD-20 surface antigen, the good long-term safety of rituximab based on the experience in oncology and rheumatology, and finally the much cheaper price of rituximab compared to newer B cell-depleting therapies. In our MS center at AUBMC, 18.4% (256/1392) of patients on DMDs are treated with rituximab, and among patients on the second/third-line therapies, 33.25% are on rituximab followed by fingolimod 29.2%, natalizumab 14.4%, and alemtuzumab 0.9%. Around 28% of our patients with PMS are treated with rituximab, usually in the early stages of the progressive phase. Due to its low yearly price which is lower than all injectables, oral therapies, and monoclonal antibodies, and in view of its good safety and efficacy profile, it has become the DMD of choice for Syrian and Palestinian refugees in Lebanon who have limited financial coverage for all MS therapies. In spite of that, most publications regarding the off-label use of rituximab in MS came from a single country, namely, Sweden, where rituximab accounts for almost 40% of all DMDs used for MS. The importance of our study is to confirm the safety and efficacy of rituximab in a different population of MS patients. Our results are similar to what has been reported by other studies. Salzer et al. [11] reported on 822 MS patients derived from the national Swedish MS registry and treated with rituximab for a mean period of 23.1 months. Rituximab induced a significant decrease in relapse rate in both RRMS (ARR = 0.0440) and PMS (ARR = 0.015–0.038) patients, similar to what we saw in our patients. In addition, rituximab effectively suppressed MRI activity with only 4.6% of patients showing enhancing lesions while on treatment as opposed to 26.2% at baseline, again similar to what we showed in our cohort. The median EDSS of their patients remained unchanged in RRMS patients and showed a statistically nonsignificant increase in PMS patients [11]. Other studies derived from the Swedish registry assessed rituximab in treatment naïve patients [12], in JC virus antibody-positive patients switching from natalizumab [13], and in comparison to first-line injectable therapies [14]. They all showed similar efficacy results when compared to the larger Swedish registry [9] and to our current study. Two smaller series of rituximab-treated MS patients were reported from the USA. Barra et al. [15] reviewed their single-center experience in 107 MS patients treated with rituximab for a mean duration of 33.2 months. In the 54 patients with RRMS, ARR on rituximab was 0.19 but no pretreatment ARR was reported. New or Gd + lesions were seen on MRI in 11% and 3% of scans, respectively. Alldredge et al. [16] reported 40 MS patients treated with rituximab in a single center for a mean duration of 2.9 years. The ARR in the 23 patients with RRMS decreased to 0.005, with 87% of RRMS and 47% of PMS patients reported to be clinically stable at the end of the follow-up period.

Rituximab was well tolerated in our patients, with the most common adverse event being infusion-related reactions all of which were mild and self-limited. The overall rate of infection was relatively low (15.7%). Two patients (2.2%) developed serious adverse events, one of which was probably unrelated to rituximab. There were no PML cases in our series, although 27 of our patients were JCV antibody-positive and 14/27 received rituximab for more than 2 years. The number of patients is of course too small to draw any conclusions regarding the risk of PML. A similar safety profile was reported in other series [11, 15, 16], with the most common adverse event being mild infusion-related reactions. In the original phase II HERMES trial [3], the main adverse events on rituximab were infusion-related reactions most of which were mild in nature, and the rate of serious adverse events and infections was similar to placebo. The long-term safety of rituximab is well established in patients with rheumatologic disorders and non-Hodgkin's lymphoma. A recent study by Vollenhoven et al. [17] reported on the long-term safety of rituximab in 1246 rheumatoid arthritis (RA) patients who received treatment for 5–11 years. The incidence of serious infections was 2.7/100 patient years with no increased risk of malignancies. To our knowledge, no cases of PML were reported in MS patients treated with rituximab. The few cases of PML reported with this medication occurred in patients with lymphoma, RA, or concurrent chemotherapeutic agents, all of which are known risk factors for this opportunistic infection [18, 19]. Hypogammaglobulinemia has been reported with chronic use of rituximab but mostly in patients with hematological malignancies. The incidence of hypogammaglobulinemia can range from 20 to 50% in patients treated for lymphoma [20] but is much lower in RA and ANCA-associated vasculitis (4% and 4.2%, respectively) [17, 21]. A similar low incidence of 3% has been reported in MS patients by Salzer et al. [11], but the drop in the aggregate serum IgG levels was slight. Significant hypogammaglobulinemia with associated infections has rarely been reported in patients with MS or neuromyelitis optica treated with rituximab and mostly as single case reports [22, 23]. It is of note that in the large series of RA patients reported by Vollenhoven et al. [17], the incidence of serious infections was similar before and after development of hypogammaglobulinemia suggesting that these patients might have an inherent high risk of developing serious infections, possibly related to their underlying disease or concomitant/previous therapies.

### 4.1. Limitations

This was a single-center retrospective study which can inherently introduce selection bias. However, all patients were captured from prospectively collected data through an established registry with a protocol that includes EDSS assessment by neurostatus-certified physicians at the center and brain MRI every 6–12 months. The sample size was relatively small, but our efficacy outcomes were similar to what has been reported in larger studies. The decrease in relapse rate on rituximab compared to pretreatment baseline could in part be due to regression to the mean, but the magnitude of the drop especially in RRMS patients reflects mostly the drug effect.

## 5. Conclusion

Rituximab was well-tolerated and effective in reducing relapse rate and stabilizing disease in relapsing-remitting and progressive MS patients in our real-world clinical practice setting. Our findings are in line with other observational studies and similar to those reported in randomized controlled trials of B cell therapies.

## Figures and Tables

**Table 1:** Baseline characteristics.

Baseline characteristics (*n* = 89)	
Age (mean, SD)	40.5 ± 12.3 years
Sex (*n*, %)	*n* = 31 (34.8%); male
	*n* = 58 (65.2%); female
Disease duration (mean, SD)	7.9 ± 6.2 years
Type of MS (*n*, %)	*n* = 59 (66.3%); RRMS
	*n* = 30 (33.7%); progressive MS
Time since onset of PMS (mean, SD)	3.0 ± 2.1 years
Baseline EDSS score (mean, SD, range)	2.25 ± 1.2 (0–6.5)
Number of relapses in previous two years (mean, SD, range)	1.6 ± 1.6 (0–8)
Time between last relapse and RTX initiation (median, range)	3 months (0–20)
Median number of RTX infusions (range)	4 infusions (2–22)
Proportion of patients with 2000 mg initial loading dose (*n*)	76.4% (*n* = 68)
Baseline MRI findings (%, *n*)	28% (*n* = 24); stable
	36% (*n* = 31); new T2 nonenhancing lesions
	36% (*n* = 31); enhancing lesions

Proportion of patients with prior DMT use (%, *n*)	11.2% (*n* = 10); treatment naïve
	34.8% (*n* = 31); one previous DMT
	30.3% (*n* = 27); 2 previous DMTs
	11.2% (*n* = 10); 3 previous DMTs
	9.0% (*n* = 8); 4 previous DMTs
	1.2% (*n* = 1); 5 previous DMTs
	2.3% (*n* = 2); 6 previous DMTs

Last DMT prior to RTX	55.7% (*n* = 44); IFN
	17.7% (*n* = 14); fingolimod
	11.4% (*n* = 9); natalizumab
	5.1% (*n* = 4); mitoxantrone
	10.1% (*n* = 8); others (AZA, mycophenolate, methotrexate, teriflunomide, cyclophosphamide)


Reasons for switching to RTX	87.3% (*n* = 69); inefficacy
	5.1% (*n* = 4); positive JCV
	2.5% (*n* = 2); adverse events
	2.5% (*n* = 2); nonavailability of other DMTs
	2.5% (*n* = 2); personal decision
RTX treatment duration (mean, SD)	22.2 ± 24.8 months

SD = standard deviation; MS = multiple sclerosis; RRMS = relapsing-remitting multiple sclerosis; PMS = progressive multiple sclerosis; EDSS = expanded disability status scale; RTX = rituximab; MRI = magnetic resonance imaging; DMT = disease-modifying therapy; IFN = interferon; AZA = azathioprine; JCV = John Cunningham virus.

**Table 2:** Adverse events and serious adverse events (safety population).

All events
Any event: *n* = 64 (71.9%)
Any event leading to discontinuation of the drug: *n* = 1 (1.1%)
PML: none
Death: none

Infusion reactions (*n* = 40); 8.7%
(i) Mild reaction during first cycle (*n* = 19)
(ii) Mild reaction during second cycle (*n* = 9)
(iii) Mild reaction during third cycle (*n* = 4)
(iv) Mild reaction during ≥ fourth cycle (*n* = 8)
Infections (*n* = 14); 15.7%
(i) Urinary tract infections: *n* = 9
(ii) Upper and lower respiratory tract infections: *n* = 3
(iii) Flu: *n* = 2
Dermatological adverse events (*n* = 4) – 4.5%
(i) Pityriasis rosea: *n* = 3
(ii) Seborrheic dermatitis: *n* = 1
Fatigue (*n* = 3); 3.4%
Laboratory abnormalities (*n* = 3); 3.4%
(i) Lymphopenia (ALC = 665): *n* = 1
(ii) Eosinophilia (7%): *n* = 1
(iii) Anemia: *n* = 1
GI (nausea, abdominal pain, bloating, flatulence): *n* = 2
Weight gain: *n* = 2
Sexual dysfunction: *n* = 2
Hip fracture: *n* = 2
Headache: *n* = 2
Arthralgia: *n* = 1
Paresthesia in fingers: *n* = 1
Hair loss: *n* = 1
Loss of appetite: *n* = 1
Urinary urgency: *n* = 1

Serious adverse events requiring hospitalizations and surgical interventions (*n* = 2); 2.2%
(i) Increase in the size of a preexisting meningioma with central cystic formation and enhancement 21 months after initiating rituximab therapy: *n* = 1
(ii) Fungal vaginal infection, vaginitis, pyoderma gangrenosum vaginalis, perianal abscess with fistula formation 38 months after initiating rituximab therapy: *n* = 1

PML = progressive multifocal leukoencephalopathy; ALC = acute lymphocytic count; GI = gastrointestinal.

## Data Availability

The data used to support the findings of this study are available from the corresponding author upon request.

## References

[1] Shwarz A., Balint B., Korporal-Kuhnke M., Jarius S., Von Engelhardt K., Furwentshes A. (2016). B cell populations discriminate between pediatric-and-adult-onset multiple sclerosis. *Neurology Neuroimmunology & Neuroinflammation*.

[2] Petreit H. F., Moeller Hartmann W., Reske D., Rubbert A. (2008). Rituximab in a patient with multiple sclerosis—effect on B cells, plasma cells and intrathecal IgG synthesis. *Acta Neurologica Scandinavica*.

[3] Hauser S. L., Waubant E., Arnold D. L. (2008). B-cell depletion with rituximab in relapsing-remitting multiple sclerosis. *The New England Journal of Medicine*.

[4] Hawker K., O'Connor P., Freedman M. S. (2009). Rituximab in patients with primary progressive multiple sclerosis: results of a randomized double-blind placebo-controlled multicenter trial. *Annals of Neurology*.

[5] Hauser S. L., Bar-Or A., Comi G. (2017). Ocrelizumab versus interferon beta-1a in relapsing multiple sclerosis. *The New England Journal of Medicine*.

[6] Montalban X., Hauser S. L., Kappos L. (2017). Ocrelizumab versus placebo in primary progressive multiple sclerosis. *The New England Journal of Medicine*.

[7] Bar-Or A., Grove R., Austin D. (2014). The MIRROR study: a randomized, double-blind, placebo-controlled, parallel-group, dose-ranging study to investigate the safety and MRI efficacy of subcutaneous ofatumumab in subjects with relapsing-remitting multiple sclerosis (RRMS) (S23.006). *Neurology*.

[8] Ellwardt E., Ellwardt L., Bittner S., Zipp F. (2018). Monitoring B-cell repopulation after depletion therapy in neurologic patients. *Neurology Neuroimmunology & Neuroinflammation*.

[9] Polman C., Reingold S., Banwell B. (2011). Diagnostic criteria for multiple sclerosis: 2010 revisions to the McDonald criteria. *Annals of Neurology*.

[10] Lublin F., Reingold S., Cohen J. (2014). Defining the clinical course of multiple sclerosis. The 2013 revisions. *Neurology*.

[11] Salzer J., Svenningngsson R., Alping P. (2016). Rituximab in multiple sclerosis: a retrospective observational study on safety and efficacy. *Neurology*.

[12] Granqvist M., Boremalm M., Poorghobad A. (2018). Comparative effectiveness of rituximab and other initial treatment choices for multiple sclerosis. *JAMA Neurology*.

[13] Alping P., Frisell T., Novakova L. (2016). Rituximab versus fingolimod after natalizumab in multiple sclerosis patients. *Annals of Neurology*.

[14] Spelman T., Frisell T., Piehl F., Hillert J. (2017). Comparative effectiveness of rituximab relative to IFN-*β* or glatiramer acetate in relapsing-remitting MS from the Swedish MS registry. *Multiple Sclerosis Journal*.

[15] Barra M., Soni D., Huy Vo K., Chitnis T., Stankiewicz J. (2016). Experience with long-term rituximab use in a multiple sclerosis clinic. *Multiple Sclerosis Journal – Experimental, Translational and Clinical*.

[16] Alldredge B., Jordan A., Jaim I., Racke M. K. (2018). Safety and efficacy of rituximab: experience of a single multiple sclerosis center. *Clinical Neuropharmacology*.

[17] Van Vollenhoven R. F., Fleischmann R. M., Furst D. E., Lacey S., Lehane P. B. (2015). Longterm safety of rituximab: final report of the rheumatoid arthritis global clinical trial program over 11 years. *The Journal of Rheumatology*.

[18] Clifford D. B., Ances B., Costello C. (2011). Rituximab-associated progressive multifocal leukoencephalopathy in rheumatoid arthritis. *Archives of Neurology*.

[19] Sikkema T., Schuiling W. J., Hoogendoorn M. (2013). Progressive multifocal leukoencephalopathy during treatment with rituximab and CHOP chemotherapy in a patient with a diffuse large B-cell lymphoma. *BMJ Case Reports*.

[20] Worch J., Makarova O., Burkhardt B. (2015). Immunreconstitution and infectious complications after rituximab treatment in children and adolescents: what do we know and what can we learn from adults?. *Cancers*.

[21] Roberts D. M., Jones R. B., Smith R. M. (2015). Rituximab associated hypogammaglobulinemia: incidence, predictors and outcomes in patients with multi-system autoimmune disease. *Journal of Autoimmunity*.

[22] Valentino P., Marnetto F., Granieri L., Copabianco M., Bertolotto A. (2016). Aquaporin-4 antibody titration in NMO patients treated with rituximab: a retrospective study. *Neurology Neuroimmunology & Neuroinflammation*.

[23] Farhat L., Dara J., Duberstein S., De A. (2018). Secondary hypogammaglobulinemia after rituximab for neuromyelitis optica: a case report. *Drug Safety - Case Reports*.

